# Home-Based Gait Interventions for Adults with Stroke: A Scoping Review

**DOI:** 10.1016/j.arrct.2025.100527

**Published:** 2025-09-24

**Authors:** Brianne Darcy, Kyle B. Reed, Stacy J.M. Bamberg, Donal Murray, Joyce Maring

**Affiliations:** a Department of Health, Human Function, and Rehabilitation Sciences, George Washington University, School of Medicine and Health Sciences, Washington, DC; b Department of Mechanical Engineering, University of South Florida, Tampa, FL; c Nitto Bend Technologies, Salt Lake City, UT

**Keywords:** Gait, Home environment, Rehabilitation, Stroke rehabilitation, Walking

## Abstract

**Objective:**

To map the peer-reviewed literature regarding the methods and outcomes of home-based gait interventions for adults with stroke.

**Data Sources:**

PubMed, Cumulative Index To Nursing & Allied Health Literature, Cochrane, Scopus, and Web of Science were searched for relevant studies based on the terms “stroke,” “gait,” “home,” and “treatment,” filtered for 2015 to 2025 publications. Searches occurred on September 12, 2024, and were updated on January 10, 2025.

**Study Selection:**

Eligibility screening was performed in 2 stages: title/abstract screening and full-text review. In each stage, articles were screened against the inclusion/exclusion criteria by 2 independent reviewers.

**Data Extraction:**

Using a customized spreadsheet, we extracted characteristics of the studies, demographic and clinical participant characteristics, gait treatment and assessment methodologies, and reported outcomes. One author extracted the data, and a second cross-checked for accuracy.

**Data Synthesis:**

The titles/abstracts of 1923 articles were screened, and 116 articles underwent full-text review. Sixty-two studies met the eligibility criteria. The studies were conducted in 22 countries and included 3008 adults with chronic or subacute stroke. Home-based rehabilitation was used as a standalone treatment in 61% of studies and paired with or immediately after another rehabilitation intervention in 39% of studies. Gait treatment approaches included technology-integrated home programs (34%), home-use devices (19%), individualized home rehabilitation (15%), self-management or coaching-based programs (13%), home rehabilitation delivered by trained nonclinicians (11%), and task-specific training variations (8%). Gait speed and endurance were the most common assessments. Measurements frequently occurred in clinical settings. At least 60% of studies within each category reported statistically significant or clinically meaningful impacts on specific gait outcomes.

**Conclusions:**

Home-based rehabilitation demonstrates global interest. Current evidence related to gait treatment is heterogeneous and shows a prevalence of technology and innovation for the home. A substantial number of preliminary studies suggest emerging treatment methods requiring robust, larger studies to determine the most beneficial treatments and contexts.

Stroke is a leading cause of disability in the United States and globally.^1^^,^^2^ Although a range of long-term health challenges may follow a stroke, one of the most debilitating consequences is impaired walking ability.^3^ After a stroke, nearly 60% of stroke survivors are unable to walk without assistance,^4^ and for many individuals, impairments such as slowed walking speed, decreased walking endurance, and altered gait patterns persist indefinitely.^3^, ^4^, ^5^, ^6^ These gait impairments can have detrimental consequences, impacting quality of life,^7^ community participation,^8^ risk of falls,^9^ and even survival.^10^^,^^11^

For stroke survivors with gait impairments, effective rehabilitation services are necessary to restore walking ability and functional independence.^12^ Although these services are typically provided in hospitals, rehabilitation facilities, or other clinical settings,^13^ rehabilitation in the home is increasingly recognized as a valuable option.^14^ Patients find home-based rehabilitation more accessible, convenient, and comfortable,^15^^,^^16^ and caregivers express high satisfaction with home-based care.^17^ Rehabilitation providers share similar positive sentiments, noting the home context improves continuity of care^18^^,^^19^ and enhances opportunities for meaningful task-based practice.^16^^,^^18^ Home-based rehabilitation may also reduce health care costs, offering potential financial benefits for patients, providers, and payors alike.^20^^,^^21^ Further, accumulating research evidence suggests that home‐based rehabilitation may yield comparable (or even superior) outcomes to clinic‐based rehabilitation.^22^ These benefits have been observed across priority rehabilitation domains, including functional independence,^17^ recovery from aphasia,^23^ and improvements in upper extremity function.^24^

Although these findings are promising, the effectiveness of home-based rehabilitation for treating gait dysfunction, a foremost priority for people with stroke^25^^,^^26^ and a key contributor to their health and well-being,^7^^,^^27^ has been underexplored in rehabilitation literature. Methods of gait treatment, such as task-specific training,^12^^,^^28^ treadmill training,^29^ functional electrical stimulation,^30^ robot-assisted therapy,^31^^,^^32^ virtual reality (VR),^33^^,^^34^ and high-intensity gait training^29^^,^^34^, ^35^, ^36^ have been shown to facilitate gait improvements in this population. However, many of these treatments involve equipment, personnel, or spatial layouts not typically available within homes.^15^^,^^37^^,^^38^ Similarly, the usage of increasingly common gait assessment technologies to measure the effects of gait treatment, such as motion capture systems, instrumented walkways, and sensor devices, may also be limited in home settings.^39^^,^^40^

Barriers to implementing gait treatment in the home were somewhat mitigated during the coronavirus disease 2019 (COVID-19) pandemic as health care systems adopted remote methods to maintain continuity of care.^15^ Advances in telerehabilitation now enable patients to connect with rehabilitation clinicians remotely, while remote therapeutic monitoring technologies facilitate oversight of home-based treatments from a distance. In parallel, technology-enhanced rehabilitation tools, such as mobile applications and wearable devices, have expanded the feasibility of adapting clinical therapies for home-use.^41^^,^^42^ Although these innovations have the potential to substantially improve access to home-based gait treatment, it remains unclear what methods are used and to what extent they drive meaningful improvements in gait function.

Although primary studies of various designs have explored home-based gait treatment after stroke, a comprehensive review summarizing the evidence and identifying research gaps is notably lacking. Therefore, this scoping review aimed to provide a comprehensive overview of the existing peer-reviewed literature on the methods and outcomes of home-delivered gait interventions for ambulatory adults with gait impairments from stroke.

## Methods

To conduct this study, we followed the scoping review methodological framework developed by the Joanna Briggs Institute Scoping Review Methodology Group.^43^ The protocol was prospectively registered with the Open Science Foundation (https://osf.io/myvnf) on August 29, 2024. Reporting of this review follows the Preferred Reporting Items for Systematic Reviews and Meta-Analyses extension for Scoping Reviews.

### Eligibility criteria

Eligibility criteria were established using the Population, Concept, Context framework.^43^

#### Population

The population included adults (aged 18y or older) with a diagnosis of stroke who resided in their homes and were ambulatory but with residual gait impairments. Studies were excluded if they contained participants under the age of 18 years, without a stroke diagnosis, or not residing in their home environments.

#### Concept

The concept explored in this review was the treatment of gait dysfunction in the adult stroke population and the associated outcomes. Gait treatment refers to a range of interventions aimed at improving the functional activity of ambulation.^44^ We further defined this concept as interventions or therapies, prescribed or delivered by rehabilitation professionals, aimed at improving a person’s walking ability or the characteristics of their walking patterns. Gait treatments could be delivered in-person or remotely, given that they aligned with the gait treatment definition.

To confirm the treatment’s intent to affect gait, we required studies to include at least 1 gait-focused outcome. A gait outcome was defined as an outcome measuring an individual’s ability to walk or how they walk.^40^^,^^45^ Following the process described by Moore et al,^46^ outcomes were not considered “gait-focused” if fewer than 75% of the items or questions involved the construct of gait. This rationale was applied to outcomes assessing multiple clinical constructs (such as transfers, balance, and activities of daily living). Studies that did not use a method of gait treatment and a gait-focused outcome were excluded from this review.

#### Context

Eligible studies explored providing gait treatment within the context of the home environment. Studies reporting gait treatment outside of the participant’s home (ie, a facility or clinical setting) were excluded. However, studies in which aspects of treatment occurred in both clinical and home settings were included if the treatment variable or the treatment components targeting gait dysfunction occurred within the participant’s home. The measurement of gait outcomes was not restricted to the home setting.

### Additional eligibility criteria

Studies of all designs were eligible for inclusion; however, studies that did not present original data (ie, review studies), were not full-text publications (ie, conference abstracts), or were not published in peer-reviewed literature were excluded. Because of the author’s language limitations, we only included studies published in English.

### Data sources and search strategy

The search strategy was based on the keywords “stroke,” “gait,” “treatment,” and “home,” and their synonyms. A research librarian assisted in developing the query strings, which were pilot tested in a single database (PubMed) and then searched across 5 databases: PubMed, Cumulative Index to Nursing & Allied Health Literature, Cochrane, Web of Science, and Scopus. Details of the search strings are provided in appendix 1. Results were filtered for publication dates in the last ten years (ie, 2015-2025) to ensure the articles represented relatively current treatment methods. The database searches were implemented on September 12, 2024, and repeated on January 10, 2025, to capture additional relevant articles published during the review period. Retrieved articles were uploaded to the program Covidence^47^ a for eligibility screening.

### Eligibility screening and study selection

Duplicate manuscripts were identified and removed within Covidence. Eligibility screening occurred in 2 stages. In the first stage, the articles’ titles and abstracts were reviewed against the prespecified inclusion and exclusion criteria. Irrelevant studies were excluded from further consideration. In the second stage, the full texts of the remaining articles were reviewed for final determination of eligibility. In each stage, articles were screened by two independent reviewers from the author team. Conflicts in eligibility decisions were resolved through discussion between the reviewers to achieve consensus or consultation with a third reviewer. To minimize authorship bias, studies authored by any of the reviewers were screened by 2 nonauthor reviewers.

### Data extraction

A customized data extraction form was created to record the data from the included studies. The form was pilot tested by the authors to ensure it comprehensively captured data relevant to the research question and the population, concept, context, and criteria.^43^

Data extraction for each study was completed by a single reviewer and cross-checked by a second reviewer. Discrepancies in data extraction were resolved by discussion between the reviewing individuals. For included studies authored by a member of the author team, the data extraction was performed and cross-checked by 2 nonauthor research team members.

### Data analysis

Data were analyzed using descriptive analysis, including frequency counts, percentages, and narrative descriptions. The analysis aimed to evaluate the following: the characteristics of the included studies, the demographic and clinical characteristics of study participants receiving home-based gait treatment, the methods and types of gait treatments provided, home-based gait assessment methodologies, and the reported outcomes of these treatments.

## Results

### Study selection

**Fig 1:**
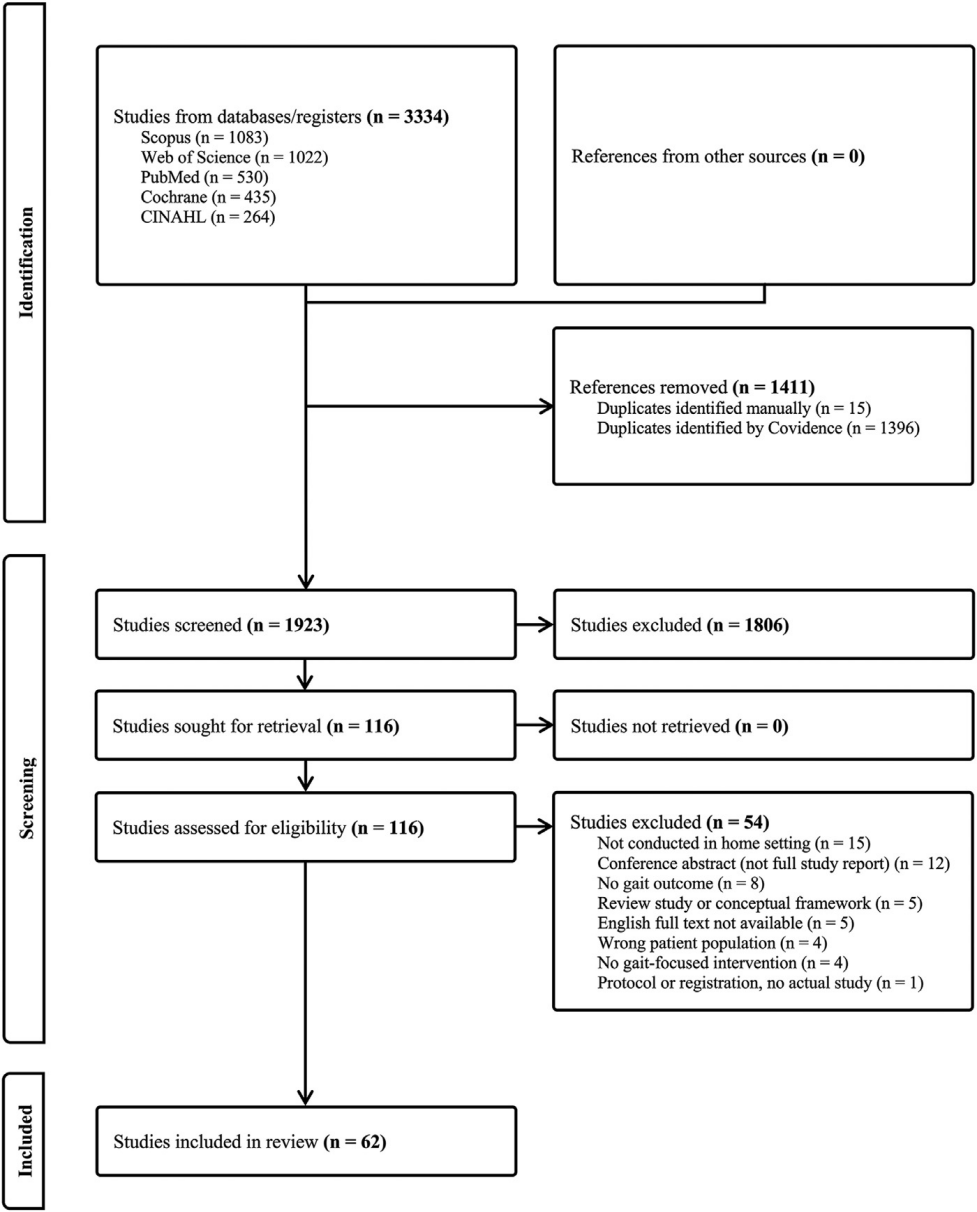
PRISMA flow diagram.

Overall, 1923 studies were screened, and 62 met the eligibility criteria for inclusion. The number of studies screened at each stage and the reason for exclusion are shown in the PRISMA flow diagram in figure 1.

### Characteristics of the included studies

**Fig 2:**
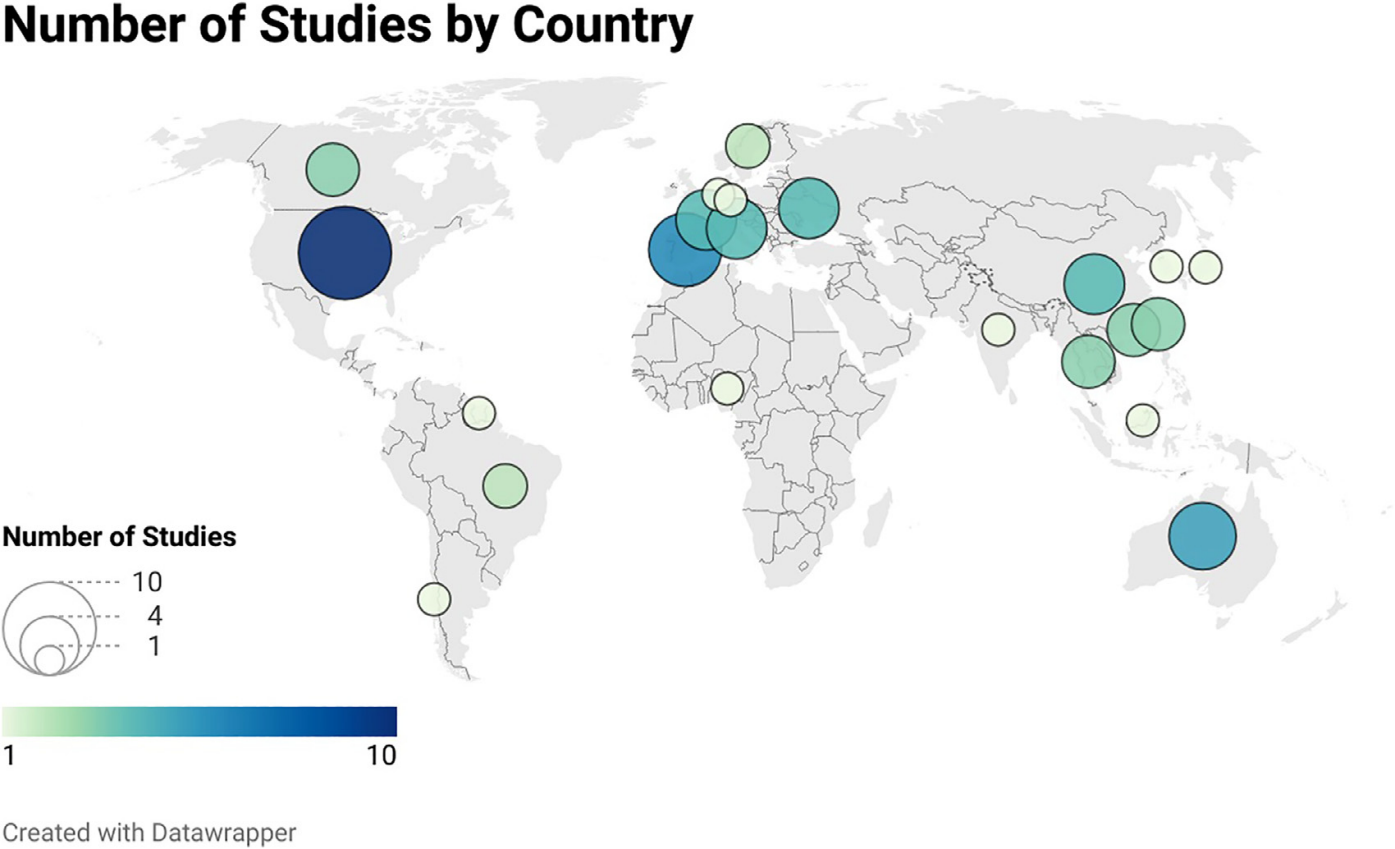
Global distribution of studies.

The 62 studies were conducted in 22 countries across all continents except Antarctica. The greatest number of studies were conducted in the United States (10 studies, 16%), followed by Spain (6 studies, 10%), Australia (5 studies, 8%), and China, France, Italy, and the United Kingdom (4 studies each, 6%). The remaining studies were from a range of countries in North and South America, Europe, Asia, and Africa. The global distribution of studies is shown in figure 2.

Thirty-seven studies (60%) compared 2 treatment groups; 32 studies (52%) were randomized controlled trials, 3 were nonrandomized controlled trials, 1 was a randomized crossover design, and one was a matched case-control study. Twenty-five studies (40%) used a single treatment group, and five of these studies (8%) were case reports or case series. Of the 62 studies, 24 (39%) were referred to as pilot, feasibility, preliminary, or proof-of-concept studies, and three studies (5%) reported follow-up results to an original study. The greatest number of studies was published in the year 2022 (11 studies, 18%), followed by the years 2021 (10 studies, 16%), 2016 (8 studies, 13%), and 2024 (7 studies, 11%).

### Participant characteristics

The studies included 3008 individuals with stroke. Of the total participants, 1809 (60%) were men, 1182 (39%) were women, and 17 (1%) were not specified. The mean age of participants was less than 55 years in six studies (10%), between 55 and 65 years in 39 studies (63%), and between 65 and 75 years in 17 studies (27%). Participants in 43 studies (69%) had a mean or median poststroke duration representing the chronic phase of stroke (at least 6 months after the stroke event), whereas 18 studies (29%) represented the subacute phase (7d to 6mo poststroke),^48^ and 1 study compared outcomes in both groups, subacute and chronic. Of the 42 studies (68%) that reported stroke type, 72% were ischemic strokes, 25% were hemorrhagic strokes, and 3% were not categorized.

### Gait treatment approaches

The studies reported various approaches to treating gait dysfunction in the home. Treatment methods were broadly grouped into 6 categories, described in table 1. In categorizing the treatments, we observed that some study methodologies contained elements of multiple treatment approaches. In such instances, categorization was matched to the treatment variable or approach under investigation using a 2-reviewer consensus.

**Table 1:** Approaches to home-based gait treatment.

Treatment Approach	No. of Studies n (%)	Description
Technology-integrated home programs*	21 (34)	Studies that employed technology-based tools, such as mobile applications (n=6), telerehabilitation systems (n=11), digital or video-based programs (n=3), and virtual, gaming, or augmented reality systems (n=9), in the treatment of gait.
Home-use devices for gait treatment*	12 (19)	Gait-focused treatment devices, including FES devices (n=3), a wearable overground gait device (n=2), home-use lower extremity robotic devices (n=2), electrode-embedded garments (n=2), orthotic or shoe lift variations (n=3), and a tDCS device (n=1).
Individualized home rehabilitation programs	9 (15)	Studies using conventional rehabilitation treatment methods within the home, combining activities such as strengthening, balance training, and functional activities tailored to the participant’s specific impairments and goals.
Self-management or coaching-based programs	8 (13)	Studies that provided self-management (n=6) or coaching programs (n=2) focused on increasing activity levels or exercise performance. While these programs were designed for participants to complete independently within their homes, supportive tools, such as monitoring or feedback, were used to encourage adherence.
Home rehabilitation delivered by trained nonclinicians	7 (11)	Studies that involved training non-clinical personnel, such as caregivers (n=5), aides (n=1), or village health workers (n=1), to perform or manage rehabilitation delivery.
Variations of task-specific training	5 (8)	Gait-specific training methodologies included progressive walking based programs (n=2), variations of auditory cueing during gait training (n=2), and implicit and explicit motor learning approaches to gait training (n=1).

Abbreviations: FES, functional electrical stimulation; tDCS, transcranial direct current stimulation.

⁎ Several studies employed multiple treatment tools; therefore, the number of studies using the various treatment approaches exceeds the total number of studies.

### Characteristics of home-based gait interventions

#### Treatment timing and duration

Thirty-eight studies (61%) investigated home-based gait treatment as a standalone treatment, independent of other rehabilitation interventions. The remaining studies delivered home-based gait training either following or paired with another form of rehabilitation. Among the 15 studies (24%) in which home-based treatment followed another rehabilitation intervention, 12 studies (19%) initiated the home-based treatment immediately after acute stroke discharge, whereas 3 studies (5%) used home-based gait treatment directly after a period of clinic-based treatment.

Of the 9 studies (15%) that paired home-based gait treatment with a concurrent form of rehabilitation, 7 studies (11%) paired the home-based treatment with a clinic-based treatment, and 2 (3%) paired the investigative home-based treatment with existing home health services.

The duration of home-based treatment ranged from 2 weeks to 12 months, with over half of the programs (33 studies, 53%) ranging between 8 and 12 weeks. The greatest number of programs (18 studies, 29% of studies) were 12 weeks in duration. When reported, the frequency and total number of treatment sessions are shown within the outcome tables below (tables 2-7).

#### Equipment used during treatment

Equipment used during home-based treatment was varied, with the majority involving technology-based tools within the treatment regimens. Tools included smartphones, laptops, and tablets (15 studies, 24%); monitoring devices (activity monitors or accelerometers, 11 studies, 18%; physiological monitoring devices, 3 studies, 5%); mobile applications (8 studies, 13%); gaming, VR, or augmented reality systems (9 studies, 15%); wearable methods of electrical stimulation (functional electrical stimulation devices or electrode-embedded garments, 7 studies, 11%); wearable overground gait or robotic devices (5 studies, 8%); and other treatment enhancements, such as metronomes and electronic exercise videos (3 studies each, 5%). Nontechnological tools included adherence diaries (11 studies, 18%) and instructional manuals (7 studies, 11%).

#### Clinic involvement in home-based treatment

Despite this review centering on the gait treatment in the home, most studies (32 studies, 52%) required participants to engage with clinical environments in some capacity. Reasons included introductory training sessions (11 studies, 18%), clinical treatment in addition to or preceding the home-based components (11 studies, 18%), equipment fitting or equipment training sessions (8 studies, 13%), or interim progress monitoring (2 studies, 3%).

#### Treatment delivery personnel

As specified in the inclusion criteria, all treatments were prescribed or delivered by rehabilitation professionals. In 43 studies (69%), the treatment was delivered by rehabilitation clinicians (physical and/or occupational therapists). Four studies (6%) involved delivery by rehabilitation researchers, and 8 studies (13%) appeared to involve a combination of clinicians and researchers.

In 7 studies (11%), rehabilitation professionals formally trained nonhealth care workers to perform or manage the in home treatment (caregivers, 5 studies, 8%; aides, 2 studies, 3%). In addition to these 7 studies, 22 additional studies (35%) involved caregivers or other family members, when available, to support treatment implementation. Their roles included providing equipment assistance, supporting treatment adherence, or receiving education on the treatment program.

#### In-Person versus remote methodologies

Interactions with care providers occurred in-person in 29 (47%) studies. In the remaining 33 studies (53%), providers used fully remote treatment protocols (ie, telehealth technologies) or a combination of in-person and remote treatment methodologies.

### Gait assessment methods

**Fig 3:**
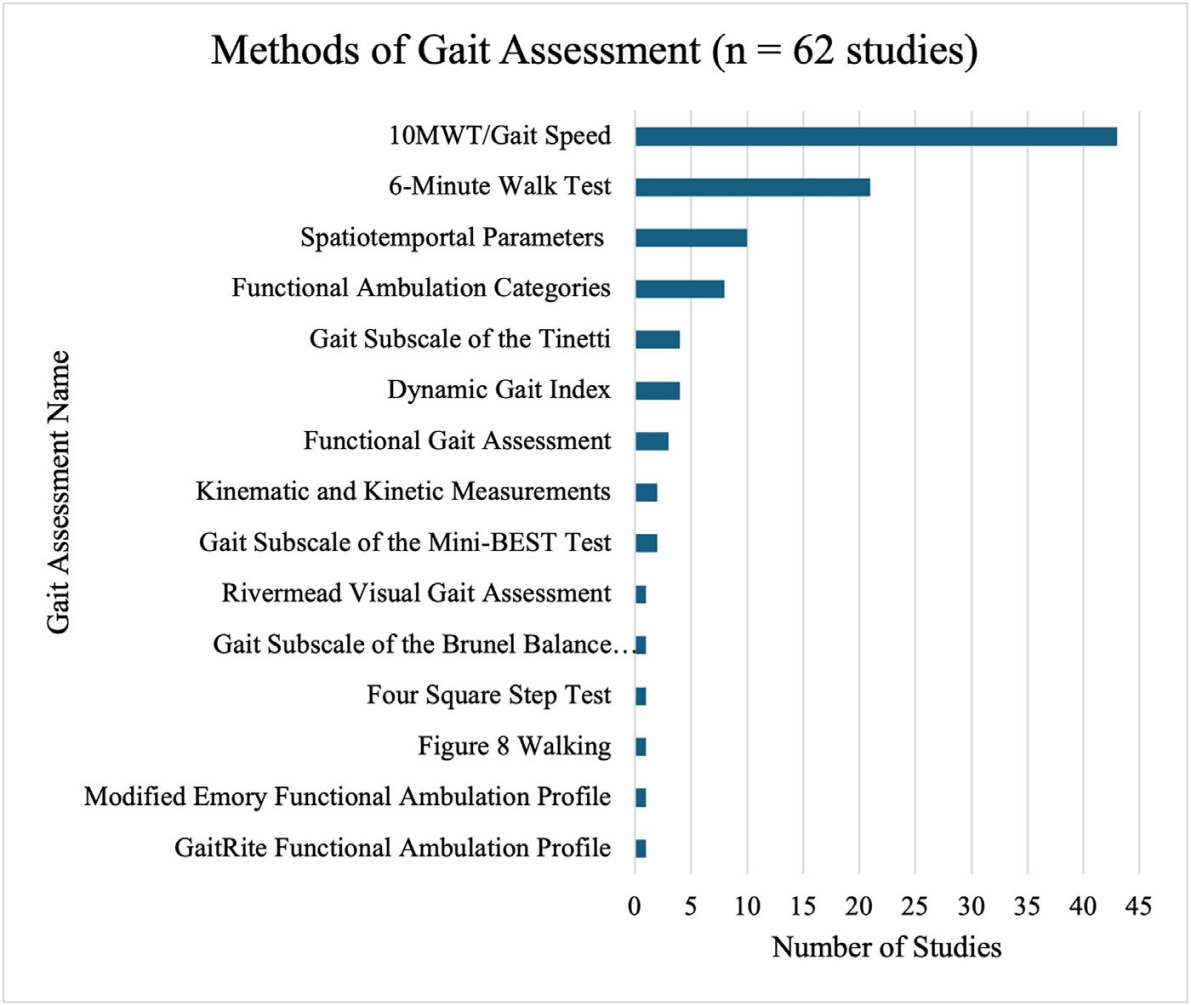
Outcomes used in the included studies.

The most frequently reported assessments were gait speed (43 studies, 69%), typically measured using the 10-meter walk test (10MWT), and walking endurance (21 studies, 34%), measured using the 6-minute walk test (6MWT). Other commonly used assessments included measurements of spatiotemporal gait parameters (10 studies, 16%), functional ambulation categories (FAC) (8 studies, 13%), the gait subscale of the Tinetti performance-oriented mobility assessment (4 studies, 6%), and the dynamic gait index (DGI) (4 studies, 6%). A brief description of each outcome is provided in appendix 2. Equipment used during gait assessment, typically to measure spatiotemporal parameters or kinematic data, included sensors (5 studies, 8%), motion capture technologies (3 studies, 5%), footprint analysis (2 studies, 3%), and instrumented walkways (1 study, 2%). Figure 3 shows the names and frequencies of gait assessments used within the studies.

Despite gait treatment occurring in the home environment, most studies (34 studies, 55%) measured the effects of gait treatment outside of the home in clinic, laboratory, hospital, or university settings. Assessment in the home was described in 16 studies (26%), and 1 study (2%) assessed gait in both home and laboratory settings. Eleven studies (18%) did not report the assessment location.

### Outcomes of home-based gait treatment

Outcomes from the 62 included studies, grouped by treatment category, are shown in tables 2-7. Each table includes a brief description of the study and population characteristics, along with the reported outcomes. Although not all studies were designed or powered to evaluate the efficacy of the gait treatment, when reported by the authors, we indicate the statistical significance of within-groups and/or between groups findings using symbols (*statistically significant; †not statistically significant). For studies that did not report statistical significance, the magnitude of the reported change is shown.

In addition, for some of the more commonly used outcomes (10MWT, 6MWT, functional gait assessment [FGA], and DGI), reported changes are compared with the outcome’s minimal clinically important difference (MCID) or minimal detectable change (MDC) value. The MCID value describes the smallest amount of change that might be considered important or meaningful to the individual.^49^ For individuals poststroke, an MCID value of 0.16 m/s has been reported for gait speed,^50^ and 34.4 m for the 6MWT.^51^ The MDC value refers to the minimal amount of change considered to exceed the measurement error of the outcome.^52^ The MDC values have been reported for the DGI at 1.9 points,^53^ and the FGA value at 4.2 points.^54^ Because MCID and MDC threshold values have not been reported for all outcomes used in these studies, these comparisons are limited to the outcomes listed.

#### Outcomes from technology-integrated home programs

Table 2 shows the outcomes reported from the 21 studies (34%) that integrated technology-based tools within their treatment paradigms. Within these studies, statistically significant within-group findings were reported for gait speed in 7 studies (33%). Additionally, individual studies (each representing [5%] of the total) reported statistically significant improvements in the following outcomes: walking endurance, figure 8 walking, the 4-square step test, the gait subscale of the Tinetti, FAC, kinematic measures of hip abduction, hip flexion, and knee flexion angles, and spatiotemporal measurements of cadence, stride length, and double support. Further, 6 studies (29%) reported improvement in gait speed beyond the MCID value of 0.16 m/s,^50^ 3 studies (14%) reported improvement in walking endurance beyond the MCID value of 34.4 m,^51^ and 1 study (5%) reported improvement in the FGA beyond the MDC value.^54^ Between groups comparisons and the direction of significance are shown within the table.

**Table 2:** Outcomes from technology-integrated home programs.

Study	Design	Population	Treatment Protocol	Gait Assessment Methods	**Outcomes**
Åkesson et al., 2025^55^	RCT (pilot)	IG (N=10) Age: 76.6± 7.1 y Chronicity: Subacute (125.6±35.0 d poststroke) CG (N=11) Age: 70.2±10.4 y Chronicity: Subacute (122.7 ± 34.7 d poststroke)	IG: Home-based training using the DISKO-tool, which included Kinect sensors, animated avatars, serious gaming, and therapist supervision using telehealth; 5 d/wk for 6 wk CG: Conventional rehabilitation in the home for 6 wk	1) 6MWT 2) Gait Subscale of the Mini-BESTest	**6MWT:** IG: <MCID† CG: <MCID† Between Groups† **Gait Subscale of the Mini BESTest:** IG: † CG: † Between Groups:†
Choukou et al, 2023^56^	Case study	N=1 Age: 55 y Chronicity: Chronic (>1-y poststroke)	VR-enabled cognitive training with tablet-based physical activity at home; 48 sessions over 12 wk	1) 10MWT	**10MWT**: >MCID
Covarrubias-Escudero et al, 2024^57^	Single-group, pre-post study	N=52 Age: 57.3±11.8 y Chronicity: Chronic (Median 21, IQR, 10.3-55 mos poststroke)	Synchronous PT sessions once/wk and asynchronous sessions 3-5 times/wk at home using a mobile app-based exercise program with a connected FES device for 10 wk	1) 10MWT	**10MWT:** <MCID*
Darcy et al, 2023^58^	Single-group, pre-post study (pilot, feasibility)	N=5 Age: Mean 72 (range, 60-80) y Chronicity: Chronic (84 mos poststroke)	Participants walked at home with the gait device on their nonparetic lower extremity 3-5 times/wk for 15-30 min over 3 mos. Supervision was provided by trained caregivers with oversight by PTs using telehealth.	1) 10MWT 2) 6MWT	**10MWT**: >MCID **6MWT**: >MCID
Davies et al, 2016^59^	Single-group study	N=5 Age: Mean, 57 (range, 42-73) y Chronicity: Chronic, 15.2 mos poststroke	Home-based program (up to 7 wk) including smart insoles for gait monitoring and access to a personalized electronic exercise library.	1) Walking Speed 2) Number of Heel Strikes 2) Symmetry	**Walking Speed**: Increased 9.8% **Number of Heel Strikes (Affected Side)**: Increased 8.8% **Symmetry:** Declined 8.5%
Ezeugwu et al, 2018^60^	Single-group feasibility study	N=34 Age: 64.6±12.5 y Chronicity: Subacute (3.5±1.1 mos poststroke)	8-wk program consisting of education, home visits, individualized treatment plans, and an activity monitoring device to reduce sedentary activity	1) Gait Speed (5MWT)	**Gait speed**: >MCID*
García-Rudolph et al, 2025^61^	Matched case-control study	IG (N=38) Age: 49.8±11.4 y Chronicity: Subacute (40±12.3 d poststroke) CG (N=38) Age: 50.9±11.8 y Chronicity: Subacute (41± 11.8 d poststroke)	IG: Telerehab program including group exercise classes, 1-on-1 PT or OT sessions, and exercise videos; ∼3.5 h/d for an average of 68 d. CG: Inpatient rehab delivered by a multidisciplinary team, 3-4 h/d for an average of 64 d	1) FAC	**FAC:** IG: 1.5-point improvement CG: 1-point improvement Between Groups: *(IG > CG)
Golla et al, 2018^62^	RCT (pilot)	IG (N=5) Age: Mean, 74.6 y Chronicity: Subacute (∼18 wk poststroke) CG (N=6) Age: Mean 73.5 y Chronicity: Subacute (∼19 wk poststroke)	IG: Balance training using the Nintendo Wii device in a clinic setting, 60 mins, 1 time/wk for 6 wk, followed by a home visit and continued Wii training at home, 30 mins, 3 times/wk for 6 wk CG: Balance training using the Otago program in a clinic setting, 60 mins, 1 time/wk for 6 wk, followed by a home visit and continued balance training at home, 30 mins, 3 times/wk for 6 wk	1) DGI	**DGI:** IG: <MDC CG: <MDC
Grau-Pellicer et al, 2020^63^	Randomized trial (pilot)	IG (N=24) Age: 63.0±11.9 y Chronicity: Chronic (18.9±27.6 mos poststroke) CG (N=17) Age: 68.5±11.5 y Chronicity: Chronic (20.6±59.7 mo poststroke)	IG: Participants received a clinic-based rehab program, a home walking program, access to an mHealth app, and a pedometer, which supervised adherence to physical activity at home. CG: Daily conventional rehabilitation for 3 mos	1) 10MWT 2) 6MWT	**10MWT:** IG: >MCID* CG: <MCID* Between Groups (strong app adherence only): *(IG > CG) **6MWT:** IG: >MCID* CG: <MCID† Between Groups (strong app adherence only): *(IG>CG)
Hsieh et al, 2018^64^	RCT	IG (N=28) Age: 58.3±12.3 y Chronicity: Chronic (12.1±6.4 mos poststroke) CG (N=28) Age: 59.3±11.9 y Chronicity: Chronic (13.0±7.1 mo poststroke)	IG: Video game-based rehabilitation using a pressure-activated electronic foot switch at home, and standard rehabilitation; 3.5 h/wk for 10 wk. CG: Standard physical therapy once/wk in a clinic setting and daily walking for 10 wk	1) 10MWT	**10MWT:** IG: >MCID* CG: <MCID† Between Groups: *(IG > CG)
Jarbandhan et al, 2022^65^	RCT (pilot, feasibility)	IG (N=20) Age: 61.6± 9.1 y Chronicity: Chronic (3.1±3.5 y poststroke) CG (N=10) Age: 62.2±9.1 y Chronicity: Chronic (4.5±3.1 y poststroke)	IG: Personalized home rehab 3 d/wk monitored using a Garmin watch and pedometer. The program was supervised for the first 4 wk and tele-supervised for the second 4 wk. CG: Usual care with no rehabilitation	1) 6MWT	**6MWT:** IG: >MCID CG: <MCID Between Groups: *(IG>CG)
Jonsdottir et al, 2021^66^	Randomized Study (pilot, feasibility)	IG (N=11) Age: 56.7±17.4 y Chronicity: Chronic CG (N=23) Age: 60.2±9.6 y Chronicity: Chronic	IG: Clinic-based VR, 3 times/wk for 4 wk, followed by home-based VR, 5 times/wk for 12 wk. CG: Clinic-based VR, 3 times/wk for 4 wk, followed by 12 wk of usual care at home	1) 10MWT 2) 2 Meter Walk Test (2MWT)	**10MWT:** IG (home period only): <MCID† CG (usual care period only): <MCID† Between Groups: † **2MWT** IG (home period only): <MCID† CG (usual care period only): <MCID† Between Groups: †
Lim et al, 2022^67^	Case Series (feasibility)	N=3 Age: Mean, 57 (range, 48-69) y Chronicity: chronic (7.9±4.4 y poststroke)	Home-based telerehab program using game-based movement priming and functional lower limb motor training; 24 sessions over 8 wk	1) 10MWT 2) FGA	**10MWT:** 1/3 participants improved >MCID **FGA:** 3/3 participants improved >MDC
Lim et al, 2021^68^	RCT	IG (N=9) Age: 70.1±6.5 y Chronicity: Chronic (62.8±8.0 mos poststroke) CG (N=8) Age: 68.6±9.1 y Chronicity: Chronic (45.1 ± 12.1 mos poststroke)	IG: Home-based rehabilitation using a combination of in-person and remote treatment; 5 times/wk for 6 wk CG: Clinic-based rehabilitation; 5 times/wk for 6 wk	1) 10MWT 2) Figure-8 Walking 3) Four Square Step Test	**1) 10MWT:** IG: <MCID* CG: <MCID* Between Groups: † **2) Figure-8 Walking:** IG: * CG: * Between Groups: *(IG > CG) **3) Four Square Step Test:** IG: * CG: † Between Groups: †
Lloréns et al, 2015^69^	RCT	IG (N=15) Age: 55.5±9.6 y Chronicity: Chronic (334.1±60.8 d poststroke) CG (N=15) Age: 55.6±7.3 y Chronicity: Chronic (316.7±49.8 d poststroke)	IG: 20 sessions with a VR-based telerehabilitation system in the home, 3 times/wk over ∼ 8 wk, and conventional rehab in the clinic 2 times/wk. CG: 20 sessions with the VR-based telerehabilitation system in the clinic, 3 times/wk over ∼ 8 wk, and conventional rehab in the clinic 2 times/wk	1) Gait Subscale of the Tinetti	**Gait Subscale of the Tinetti:** IG: * CG: * Between Groups:†
Lu et al, 2024^70^	Single-group, pre-post study	N=12 Age: 63.8±12.8 y Chronicity: Chronic (41.8 ± 33.4 mos poststroke)	Personalized exercise program delivered through a home training system, which used Kinect sensors to reconstruct a skeletal model providing real-time motor and visual feedback; 20-40 min sessions, 3 times/wk for 800 mins total	1) FAC 2) Kinematic Data of Joint Angles	FAC:* Kinematic Data of Joint Angles:*(hip abduction, hip flexion, and knee flexion angles)
Podury et al, 2021^71^	Single-group, longitudinal (pilot)	N=13 Age: Median 61 (IQR, 52–65.5) y Chronicity: Subacute (Median, 129; IQR, 52-486) d poststroke	Upper and lower extremity training using functional games and stroke education, conducted at home using a telerehabilitation system, 1 h, 6 d/wk, for 12-wk	1) 10MWT	**10MWT**: <MCID*
Salgueiro et al, 2022^72^	RCT (preliminary)	IG (N=15) Age: 57.3±14.4 y Chronicity: Chronic (4.6±3.4 y poststroke) CG (N=15) Age: 64.5±9.4 y Chronicity: Chronic (4.1 ± 4.4 y poststroke)	IG: Conventional therapy 2 times/wk for 12 wk and usage of a telerehabilitation app for home-based core-stability exercises CG: Conventional therapy, 2 times/wk for 12 wk	1) 10MWT 2) Gait Parameters	**10MWT:** IG: † CG: † Between Groups: † **Gait Parameters:** IG: All † except double support % (less affected limb) was* CG: All† Between Groups: All † except double support % (less affected limb) was* (IG>CG)
Salgueiro et al, 2022^73^	Controlled trial (pilot, feasibility)	IG (N=20) Age: 71.6±10.7 y Chronicity: Subacute (∼ 5-wk from hospital discharge after stroke) CG (N=29) Age: 70.7±14.1 y Chronicity: Subacute (∼ 5-wk from hospital discharge after stroke)	IG: Clinic-based therapy (avg of 2.5 sessions/wk) and usage of a telerehabilitation app for home-based core-stability exercises CG: Clinic-based therapy (avg of 2.5 sessions/wk)	1) Gait speed 2) Gait subscale of the Brunel Balance Assessment (BBA) 3) Cadence	**Gait speed^‡^:** IG: 0.79 m/s reduction compared to reference values CG: 0.63 m/s reduction compared to reference values **Gait subscale of the BBA:** IG:† CG:† Between Groups:† **Cadence‡:** IG: 26 fewer steps/min compared with reference values CG: 30 fewer steps/min compared with the reference values ^‡^Analysis only on 2 individuals from each group
Wang et al, 2024^74^	RCT	IG (N=40) Age: 63.0±3.1 y Chronicity: Subacute (39.1 ± 4.1 d poststroke) CG (N=40) Age: 63.01±3.0 y Chronicity: Subacute (39.1±4.2 d poststroke)	IG: Personalized home exercise program and walking training using app-based training videos and a wearable gait monitoring device; 30 min, 2 times/d, 6 d/wk for 12 wk. CG: Routine posthospital follow-up for 12 wk, including a home rehab prescription. Once/mo follow-up was encouraged	1) 10MWT 2) Stride Length 3) Cadence	**10MWT:** IG: >MCID* UG: <MCID* Between Groups: *(IG > CG) **Stride Length:** IG: * CG: * Between Groups: *(IG > CG) **Cadence:** IG: * CG: * Between Groups: *(IG > CG)
Yang et al, 2022^75^	RCT	IG (N=16) Age: 64.6±9.9 y Chronicity: Chronic (3.7 ± 2.7 y poststroke) CG (N=23) Age: 62.8±13.8 y Chronicity: Chronic (3.4±2.7 y poststroke)	IG: Home-based sessions using an AR Rehabilitation System with human and virtual trainers; 20 sessions (120-mins), 2–5 sessions/wk CG: Clinic-based sessions using an AR Rehabilitation System with human and virtual trainers; 20 sessions (120-min), 2–5 sessions/wk	1) FAC	**FAC:** IG: † CG: † Between Groups: †

Abbreviations: 2MWT, 2-minute walk test; 5MWT, 5-meter walk test; 6MWT, 6-minute walk test;10MWT, 10-meter walk test; AR, augmented reality; avg, average; BBA, Brunel Balance Assessment; CG, comparison group; DGI, dynamic gait index; FAC, functional ambulation categories; FGA, functional gait assessment; IG, intervention group; IQR, interquartile range; m/s, meters per second; MCID, minimal clinically important difference; MDC, minimal detectable change; NS, not significant; PT, physical therapist; RCT, randomized controlled trial; SS, statistically significant; VR, virtual reality.

⁎ Statistically significant (p<0.05)

† Not statistically significant (p≥0.05)

#### Outcomes from home-use devices for gait treatment

Table 3 shows the outcomes from the 12 studies (19%) featuring home-use devices for gait treatment. Statistically significant improvements were reported for walking speed (6 studies, 50%), walking endurance (2 studies, 17%), various spatiotemporal gait parameters (2 studies, 17%), and FAC (2 studies, 17%). Additionally, one study (8%) reported statistically significant improvement in the Rivermead visual gait assessment, FGA, kinematic joint parameters, the gait subscale of the Tinetti performance-oriented mobility assessment, and the DGI. Further, 5 studies (42%) reported gait speed improvement beyond the MCID,^50^ and one study each (8%) reported improvement beyond the MDC for the FGA^54^ and DGI.^53^

**Table 3:** Outcomes from home-use devices for gait treatment.

**Study**	**Design**	Population	Treatment Protocol	Gait Assessment Method	Outcomes
Bethoux et al, 2015^76^	RCT (follow-up)	IG (N=242) Age: 63.9±11.3 y Chronicity: Chronic (6.9±6.4 y) CG (N=253) Age: 64.3±12.0 y Chronicity: Chronic (6.9±6.6 y poststroke)	IG: FES used during community and household ambulation for 12 mons CG: AFOs used during community and household ambulation for 12 mons	1) 10MWT 2) 6MWT 3) GaitRite functional ambulation profile 4) Modified Emory functional ambulation profile	**10MWT:** IG=>MCID* CG=>MCID* Between Groups=† **6MWT:** IG≤MCID* CG≤MCID† Between Groups=† **GaitRite Functional Ambulation Profile:** IG=† CG=† Between Groups=† **Modified Emory Functional Ambulation Profile:** IG=† CG=† Between Groups=†
Bhalerao et al, 2016^77^	RCT	IG (N=13) Age: 54.5±7.5 y Chronicity: Chronic (> 6 mo poststroke) CG (N=14) Age: 54.4±7.1 y Chronicity: Chronic (> 6 mo poststroke)	IG: 1 cm shoe-raise worn on the unaffected side while ambulating at home and during a gait-focused motor relearning program in a clinic setting (60 min, 6 times a wk for 4 wk). CG: Same as above (without shoe lift)	1) 10MWT 2) Spatiotemporal gait parameters 3) Rivermead visual gait assessment	**10MWT:** IG: >MCID* CG: >MCID* Between Groups: *(IG > CG) **Spatiotemporal Gait Parameters:** IG: All assessed parameters * except angle of toe-out (nonparetic) were † CG: All assessed parameters * except angle of toe-out (nonparetic) were † Between Groups: All *(IG > CG) except angle of toe-out (paretic and nonparetic) were † **3) Rivermead Visual Gait Assessment:** IG: * CG: * Between Groups: †
Darcy et al, 2024^78^	Single-group, pre-post study with long-term follow-up	N=18 Age: 57±8 years Chronicity: Chronic (60±0 mo poststroke)	Participants walked in their homes with the gait device on their nonparetic lower extremity; 30 min, 3 times/wk for 4 wk	1) 10MWT	**10MWT**: >MCID*
David et al, 2022^79^	Case study	N=1 Age: 22 y Chronicity: Chronic (4 y poststroke)	Home FES device use daily for 6 mo and during PT sessions once/wk	1) 10MWT 2) 6MWT (Each test was performed with 1) No orthosis, 2) With AFO, and 3) With FES	**10MWT**: No Orthosis: >MCID With AFO: <MCID With FES: >MCID **6MWT**: No orthosis: <MCID With AFO: <MCID With FES: <MCID
Housley et al, 2016^80^	Single-group, pre-post study	N=10* Age: 70.6±12.7 y Chronicity: Chronic (19.4 mo poststroke) *Lower extremity device users only	Robotic-assisted lower extremity rehab at home using game-like training programs; 2 h/d for 3 mo with remote follow-up	1) 10MWT 2) 6MWT	**10MWT**: <MCID† **6MWT**: <MCID†
Huizenga et al, 2021^81^	Single-group, pre-post study	N=21 Age: 55.7±8.6 y Chronicity: Chronic (61.3±64.9 mos poststroke)	Participants ambulated in their homes with the gait device on their nonparetic lower extremity; 30 min, 3 times/wk for 4 wk	1) 10MWT 2) FGA	**10MWT:** >MCID* FGA: >MDC*
Lopez-Rosado et al, 2019^82^	Case series (pilot)	N=15 Age: 56.5±7.8 y Chronicity: Chronic (8.2 + 4.4 y poststroke)	Home-based sensory amplitude electrical stimulation (SES) was delivered using a Silver-Thera sock electrode on the more involved foot. Participants performed standing and mobility activities while receiving SES for at least 30 min, twice a d, 5 ds/wk, for 6 wk.	1) 10MWT	**10MWT**: <MCID*
Mao et al, 2022^83^	RCT	IG (N=16) Age: 52.3±9.2 y Chronicity: Chronic (19.8±5.9 mos poststroke) CG (N=15) Age: 54.8±10.6 y Chronicity: Chronic (19.7±4.1 mos poststroke)	IG: tPNS device use during ambulation at home and in the community for increasing durations over 3 wk. CG: Home rehabilitation program guidance including walking and gait exercises over 3 wk	1) Gait Speed 2) Spatiotemporal Parameters 3) Kinematic and Kinetic Data	**Gait Speed:** IG: <MCID* CG: <MCID† Between Groups: † **Spatiotemporal Parameters:** IG: *(opposite foot off, double support, and stride length) CG: † Between Groups: *(opposite foot off and opposite foot contact; IG > CG) **Kinematic and Kinetic Data:** IG: *(ankle angle in the transverse plane, foot progression angle in the frontal and transverse planes, ankle transverse peak during stance and swing phases, and maximum foot progression peak in the frontal and transverse planes) CG: † Between Groups: *(maximum foot progression peak in the sagittal plane; IG > CG)
Palmcrantz et al, 2020^84^	Single-group, pre–post study	N=20 Age: 58.1±12.9 y Chronicity: Chronic (67.2±44.3 mo poststroke)	Participants wore the Molli suit, which stimulates the antagonist of spastic muscles at home, for 60 min, every second d, for 6 wk (21 sessions)	1) 10MWT 2) 6MWT	**10MWT**: <MCID† **6MWT**: <MCID†
Paula et al, 2024^85^	RCT	IG (N=24) Age: Median 63.5 (IQR 60-72) y Chronicity: Chronic (Median 6.5, IQR 4.2-9 mo poststroke) CG (N=23) Age: Median 62 (IQR 55-69) y Chronicity: Chronic: (Median 6; IQR, 3-10 mon poststroke)	IG: Daily fixed AFO use and progressive home mobility tasks, 30 min, 5 times/wk for 4 wk. CG: Daily articulated AFO use and progressive home mobility tasks, 30 min, 5 times/wk for 4 wk.	1) Gait subscale of the Tinetti 2) FAC	**Gait subscale of the Tinetti:** IG: † CG: * Between Groups: *(CG > IG) **FAC:** Fixed AFO Group: * Articulated AFO Group: * Between Groups: *(CG > IG)
Prathum et al, 2022^86^	RCT	IG (N=12) Age: 58.7±3.7 y Chronicity: Chronic (15.5±2.6 mo poststroke) CG (N=12) Age: 56.8±3.6 y Chronicity: Chronic (16.3±3.3 mo poststroke)	IG: 20-min active dual-tDCS at home followed by 1-h exercise, 3 times/wk for 4 wk CG: 20-min sham tDCS at home followed by 1-h exercise, 3 times/wk for 4 wk	1) Walking speed (6-meter walk test)	**Walking speed:** IG: <MCID† CG: <MCID† Between Groups::†
Wright et al, 2021^87^	RCT	IG (N=16) Age: 59.6±10.1 y Chronicity: Chronic (31±19 mo poststroke) CG (N=18) Age: 65.1±10.1 y Chronicity: Chronic (32±21 mo poststroke)	IG: Wearable robotic knee orthosis during walking and exercise in the home for at least 30 min/d and weekly, clinic-based physical therapy for 10 wk CG: Recommendation for 30-min of physical activity/d and weekly clinic-based physical therapy for 10 wk	1) 6MWT 2) FAC 3) DGI	**6MWT:** IG: <MCID* CG: <MCID† Between Groups: *(IG > CG) **FAC:** IG: * CG: † Between Groups: *(IG > CG) **DGI:** IG: >MDC* CG: <MCID† Between Groups: *(IG > CG)

Abbreviations: 6MWT, 6-minute walk test;10MWT, 10-meter walk test; AFO, ankle foot orthosis; CG, comparison group; DGI, dynamic gait index; FAC, functional ambulation categories; FES, functional electrical stimulation; IG, intervention group; IQR, interquartile range; NS, not significant; PT, physical therapy; RCT, randomized controlled trial; tDCS, transcranial direct current stimulation; tPNS, transcutaneous peripheral nerve stimulation; SS, statistically significant.

⁎ Statistically significant (p<0.05)

† Not statistically significant (p≥0.05)

#### Outcomes from individualized home rehabilitation programs

Table 4 shows the outcomes from the 9 studies (15%) that implemented individualized home-based rehabilitation programs. Statistically significant improvements were reported for walking speed (2 studies, 22%), walking endurance (3 studies, 33%), FAC (1 study, 11%), and the gait subscale of the Tinetti performance-oriented mobility assessment (1 study, 11%). Additionally, 2 studies (22%) reported gait speed improvement beyond the MCID,^50^ and 4 studies (44%) reported improved walking endurance beyond the MCID.^51^

**Table 4:** Outcomes from individualized home rehabilitation programs.

Study	Design	Population	Treatment Protocol	Gait Assessment Methods	Outcomes
Brouwer et al, 2018^88^	RCT	IG (N=51) Age: 62.7±1.9 y Chronicity: Chronic (6 mo - 1 y after inpatient discharge) CG (N=52) Age: 62.1±1.8 y Chronicity: Chronic (6 mo-1 y after inpatient discharge)	IG: Home-based "tune-up" interventions at 6- and 12-mo postdischarge, consisting of 6 individualized 1-h sessions, 3 times/wk, for 2 wk. CG: No treatment provided (only assessments)	1) 6MWT	**6MWT:** IG: <MCID (6 mo); >MCID (12 mo) CG: >MCID (6 mo); >MCID (12 mo) Between Groups: †
Chen et al, 2021^89^	RCT	IG (N=59) Age: 55.4±6.8 y Chonicity: Subacute (3.4±0.8 mo poststroke) CG (N=62) Age: 56.4±6.1 y Chronicity: Subacute (3.2±0.8 mo poststroke)	IG: Individually tailored home rehab program conducted by an advanced practice RN; 30-min sessions progressively decreased from 3 times/wk to every other month over a 12-month period. CG: Rehabilitation manual and routine medical follow-up	1) 10MWT 2) Step Size	**10MWT:** IG: >MCID CG: >MCID Between Groups: *(IG > CG) **Step Size:** IG: Step size increased 0.4 m CG: Step size increased 0.2 m Between Groups: *(IG > CG)
Chung et al, 2020^90^	RCT (pilot)	IG (N=27) Age: 66.9±14.0 y Chronicity: Subacute (41.3±16.5 d poststroke) CG (N=29) Age: 72.5±15.5 y Chronicity: Subacute (37.9±14.2 d poststroke)	IG: Video home exercise program accessed using a QR code and smart device; Doses were 10-30 min/d for 3 mo. CG: Home exercise program pamphlet with photographs and instructions; Doses were 10-30 min/d for 3 mo	1) Modified functional ambulation categories (mFAC)	**mFAC** IG: * CG: * Between Groups: *(IG > CG)
Dunn et al, 2017^91^	Single-group, long-term follow-up study (follow-up to Marsden 2017)	N=20 Age: 60.1±19.2 y Chonicity: Subacute (5.3±3.5 mo poststroke)	Individually tailored, home and community-based exercise program for 12 wk (12-mo outcomes reported)	1) 10MWT 2) 6MWT	**10MWT**: >MCID* 6MWT: >MCID*
Ewah et al, 2024^92^	Case report	N=1 Age: 63 y Chronicity: Chonic (∼1-y poststroke)	Home-based rehab activities, such as progressive resistance and gait/balance training; 2-h sessions, 3 times/wk, for 8 wk	1) Gait Subscale of the Tinetti	**Gait Subscale of the Tinetti**: 4-point improvement
López-Liria et al, 2016^93^	Non-randomized comparative trial	IG (N=78) Age: 74.1±10.8 y Chronicity: Presume subacute (after acute stroke discharge) CG (N=67) Age: 64.5±11.8 y Chronicity: Presume subacute (following acute stroke discharge)	IG: Individualized home rehab to minimize stroke functional impairments; 2-3 times/wk for an avg of 21 sessions CG: Individualized hospital based rehab to minimize stroke functional impairments; 2-3 times/wk for an avg of 29 sessions	1) Gait Subscale of the Tinetti	**Gait Subscale of the Tinetti:** IG: * CG: * Between Groups: *(IG > CG)
Marsden et al, 2016^94^	Pilot controlled trial (not randomized)	IG (N=10) Age: 54.4±22.2 y Chonicity: Subacute (5.6±5.3 mo poststroke) CG (N=10) Age: 62.0±16.8 y Chonicity: Subacute (3.8±1.2 mo poststroke)	IG: 12-wk individually tailored home- and community-based exercise program using a whole-d approach to being more active. CG: Usual care consisting of required medical or therapy appointments. No additional treatment provided	1) 10MWT 2) 6MWT	**10MWT**: IG: <MCID CG: <MCID Between Groups: † **6MWT**: IG: >MCID CG: <MCID Between Groups: *(IG > CG)
Rose et al, 2017^95^	Secondary analysis (of RCT)	IG (N=118) Age: 61.4±13.0 y (total sample) Chonicity: Subacute (2-mo poststroke) CG/Late (N=112) Age: 61.4±13.0 y (total sample) Chonicity: Chonic (6-mo poststroke) CG/Early (N=117) Age: 61.4±13.0 ys (total sample) Chonicity: Subacute (2-mo poststroke)	IG: Individualized exercise program in the home; 90-mins, 3 times/wk over 12-16-wk CG/Late: Task-specific walking training on a treadmill with partial body weight support and individualized progression; 90-mins, 3 times/wk over 12-16-wk (beginning 6-mo poststroke) CG/Early: Task-specific walking training on a treadmill with partial body weight support and individualized progression; 90-mins, 3 times/wk over 12-16-wk (beginning 2-mo poststroke)	1) 10MWT 2) 6MWT	**10MWT (baseline to 12 sessions):** IG (Moderate): <MCID* IG (Severe): <MCID* CG/Late (Moderate): <MCID* CG/Late (Severe): <MCID* CG/Early (Moderate): >MCID* CG/Early (Severe): <MCID* 6MWT (baseline to 12 sessions): IG (Moderate): >MCID* IG (Severe): >MCID* CG/Late (Moderate): <MCID* CG/Late (Severe): <MCID* CG/Early (Moderate): >MCID* CG/Early (Severe): <MCID*
Saggini et al, 2021^96^	Single-group, pre-post study	N=73 Age: Mean 70 (Range 59–77) y Chonicity: Chonic (stroke occurred at least 6 mo before)	Home rehabilitation, 2 times/wk for 40 sessions	1) 10MWT 2) 6MWT	**10MWT**: <MCID† **6MWT**: <MCID*

⁎ Statistically significant (p<0.05)

† Not statistically significant (p≤0.05) Abbreviations: 6MWT, Six-Min Walk Test;10MWT, Ten-Meter Walk Test; CG, comparison group; h, hour; IG, intervention group; IQR, interquartile range; m/s, meters per second; MCID, minimal clinically important difference; mFAC, Modified Functional Ambulation Categories; mins, min; NS, not significant; RCT, randomized controlled trial; SS, statistically significant; y, ys

#### Outcomes from self-management or coaching-based programs

Table 5 reports the outcomes from self-management or coaching-based programs. Of the 8 studies, statistically significant findings were reported for gait speed (4 studies, 50%), walking endurance (3 studies, 38%), and cadence (1 study, 13%). Additionally, 4 studies (50%) reported clinically meaningful improvements in walking endurance beyond the MCID value.^51^

**Table 5:** Outcomes from self-management or coaching-based programs.

Study	Design	Population	Treatment	Gait Assessment Method	Outcomes
Caetano et al, 2023^97^	Single-group, pre-post study (feasibility)	N=20 Age: 64±11 y Chronicity: subacute (3.8± 0.1 mo poststroke)	Self-management program focused on increasing physical activity, delivered by a PT in 6, 1 h home-based sessions over 3 mo, and performed independently between sessions	1) Gait speed (8MWT) 2) 6MWT	**Gait Speed (8-MWT)**: <MCID **6MWT**: >MCID
Guediri et al, 2022^98^	RCT	IG (N=27) Age: 62.1±13.2 y Chronicity: subacute (75.3±40.0 d poststroke) CG (N=20) Age: 56.4±12.3 y Chronicity: subacute (91.6±51.7 d poststroke)	IG: Coaching-based program including education and physical activity monitoring with weekly phone calls and home visits every 3 wk for 6 mo CG: Medical appointments at 1 and 6 mo after hospital discharge and general poststroke activity information	1) 6MWT	**6MWT:** IG: >MCID^ee^ CG: <MCID† Between Groups: *(IG > CG)
Mandigout et al, 2021^99^	RCT	IG (N=41) Age: Median,63 (IQR, 12) y Chronicity: subacute (Median 2.3, IQR 1.6 mo poststroke) CG (N=42) Age: Median, 58 (IQR, 24) y Chronicity: subacute (Median, 2.4, IQR, 1.6 mo poststroke)	IG: Individualized program of physical activity coaching, including education, activity selection, goal setting, monitoring with activity tracker, and home visits for 6 mo. CG: Usual care, which may have included medical appointments and outpatient physical therapy if needed	1) 6MWT 2) mFAC	**6MWT:** IG: >MCID* CG: >MCID† Between Groups: † **mFAC:** IG: 1 category improvement CG: No change Between Groups: *(IG > CG)
Nakayama et al, 2016^100^	Single-group, pre-post study	N=6 Age: Mean, 58.7 (range, 50-66) y Chronicity: Chronic (range, 9-18 mo poststroke)	Self-managed home exercise program consisting of 2 sets of 3-min standing on a slant board; 3 times/d for 30 d	1) 10MWT 2) Number of steps during the 10MWT (cadence)	**10MWT**: <MCID* Number of steps during the 10MWT (cadence): *(decrease)
Pradines et al, 2019^101^	RCT	IG (N=12) Age: 57±11 y Chronicity: Chronic (10±9 y poststroke) CG (N=11) Age: 55±13 y Chronicity: Chronic (8±5 y poststroke)	IG: Conventional therapy as prescribed by a physician with a guided self-rehabilitation contract consisting of daily, high-load, home self-stretching exercises and biwkly home therapy visits for 12 mo CG: Therapy as prescribed by a physician	1) 10MWT	**10MWT**: IG: <MCID CG: <MCID Between Groups: *(IG > CG)
Preston et al, 2017^102^	Single-group, pre-post study (feasibility)	N=20 Age: 68±12 y Chronicity: subacute (16±7 d poststroke)	Self-management program incorporating education, goal setting, barrier identification, self-monitoring, and feedback delivered in 5, 60-min home-based sessions over 3 mo	1) 10MWT 2) 6MWT 3) Stride Length	**10MWT**: <MCID* 6MWT: >MCID* Stride Length: †
Scrivener et al, 2021^103^	Single-group, pre-post study (feasibility)	N=14 Age: 57±14.2 years Chonicity: Chronic (35.3±30.9 mo poststroke)	Supervised treatment in the clinic, 30 mins, 1 time/wk for 4 wk, followed by 30 mins of self-directed treatment, 3 times/wk at home	1) Gait Speed (5MWT)	**Gait Speed (5MWT)**: <MCID*
Vecchio et al, 2017^104^	Single-group, pre-post study	N=10 Age: 55.4±16.2 y Chronicity: Chronic (28.7±10.6 mo poststroke)	Home-based self-rehabilitation treatment consisting of active ankle dorsiflexion movements; 12 min, 2 sessions/d, 3 times/wk, for 3 mo	1) 10MWT	**10MWT:** <MCID*

Abbreviations: 5MWT, 5-meter walk test; 8MWT, 8-meter walk test; 10MWT, 10-meter walk test; CG, Comparison Group; IG, Intervention Group; IQR, interquartile range; MCID, minimal clinically important difference; mFAC, modified functional ambulation categories; NS, Not Significant; RCT, randomized controlled trial; SS, statistically significant.

⁎ Statistically significant (p<0.05)

† Not statistically significant (p≥0.05)

#### Outcomes from home rehabilitation delivered by trained nonclinicians

Table 6 shows the outcomes of the seven studies that involved training nonclinicians to support the delivery of home rehabilitation programs. Statistically significant improvements were reported for gait speed (3 studies, 43%), walking endurance (2 studies, 29%), and the gait subscale of the mini-BESTtest (1 study, 14%). Additionally, 2 studies (29%) reported gait speed improvement greater than the MCID.^50^

**Table 6:** Outcomes from home rehabilitation delivered by trained nonclinicians.

Study	Design	Population	Treatment Protocol	Gait Assessment Method	Outcomes
Chinchai et al, 2017^105^	Single-group, pre-post study	N=27 Age: 31-40 y: 1 (3.7%); 41-50 y: 1 (3.7%); 51-60 y: 8 (29.7%); >60 y: 17 (62.9%) Chronicity: chronic, <12 mo poststroke: 1 (3.7%), 1-2 y poststroke: 6 (22.2%), >2 y poststroke: 20 (74.4%)	In home rehabilitation delivered 1 h, 1 time/wk, for 8 wk by village health volunteers trained in rehab delivery by PTs and OTs	1) 10MWT	**10MWT**: <MCID*
Feldman et al, 2022^106^	RCT (pilot, feasibility)	IG (N=30) Age: 66.0±13.0 y Chronicity: subacute (50.0±22.9 d poststroke) CG (N=30) Age: 65.2±13.5 y Chronicity: subacute (45.1±27.8 d poststroke)	IG: 30-d intervention in which home health aides, trained by PTs as “peer coaches,” mentored the frontline aides of individuals recovering from stroke to increase their mobility through greater adherence to exercise regimens. CG: Usual care home health services	1) Gait Speed (4MWT)	**Gait Speed:** IG: <MCID CG: <MCID
Kerdsawatmongkon, 2023^107^	Matched randomized trial	IG (N=9) Age: 66.4±6.5 y Chronicity: chronic (Median, 24; IQR, 12.5-42 mo poststroke) CG (N=9) Age: 62.6±7.0 y Chronicity: Chronic (Median, 20; IQR, 6.5-31 mo poststroke)	IG: Home-based boxing, balance, and trunk exercise training; 3 times/wk for 6 wk (with increased CG responsibility and decreased therapist supervision over the 6-wk period) CG: Home-based balance and trunk exercise training; 3 times/wk for 6 wk	1) Gait Subscale of the Mini-BESTest	**Gait Subscale of the Mini-BESTest:** IG: * CG: * Between Groups: †
Nordin et al, 2019^108^	RCT	IG (N=45) Age: Median, 60 (IQR, 53-68) y Chronicity: Chronic (median, 16 mo; IQR, 9.5-27.5 mo poststroke) CG (N=46) Age: Median, 59.5 (IQR, 52-66.5) y Chronicity: Chronic (Median 12, IQR, 8.2–17.2 mo poststroke)	IG: Trained caregivers delivered a home-based rehabilitation program focused on task-oriented, domestic, and cognitive activities for 12-wk CG: Therapist-delivered group therapy in a hospital rehabilitation department; 2 h sessions, once/wk for 12 wk	1) 10MWT	**10MWT:** IG: >MCID* CG: >MCID* Between Group: †
van den Berg et al, 2016^109^	Proof-of-concept (RCT)	IG (N=20) Age: 64.7±19.5 y Chronicity: subacute (20±11.5 d poststroke) CG (N=32) Age: 70.1±12.4 y Chronicity: subacute (13.9±7.9 d poststroke)	IG: Caregiver-mediated training program supported by a customized exercise app and telerehabilitation; 30 min, 5 times/wk for 8 wk CG: Usual rehabilitation care following the Australian clinical guidelines for stroke management	1) 10 MWT	**10MWT:** IG: >MCID CG: >MCID Between Groups: †
Wang et al, 2015^110^	RCT	IG (N=25) Age: 62.0±9.5 y Chronicity: Chronic (median, 18; IQR, 11.5-32 mo poststroke) CG (N=26) Age: 65.4±10.6 y Chronicity: Chronic (median,18.5; IQR, 8.75-31.75 mo poststroke)	IG: 12-wk individualized home rehab overseen by trained caregivers. PTs visited the participant’s home weekly for training and follow-up. CG: Weekly visits or phone calls by therapists for 12 wk with no specific rehab guidance given	1) 10MWT 2) 6MWT	**10MWT:** IG: <MCID* CG: <MCID† Between Groups: *(IG > CG) **6MWT:** IG: <MCID* CG: <MCID† Between Groups: *(IG > CG)
Zhang et al, 2022^111^	RCT (pilot)	IG (N=13) Age: 51.3±8.6 y Chronicity: Chronic (10.3±3.8 mo poststroke) CG (N=12) Age: 53.3±5.8 y Chronicity: Chronic (10.9±2.1 mo poststroke)	IG: Video-based sessions with participants and caregivers focused on caregiver coaching for reaching poststroke goals; weekly, for 3 mo CG: Video-based sessions with the participant and caregiver focused on poststroke goals (without caregiver coaching); weekly, for 3 mo	1) 6MWT	**6MWT:** IG: <MCID* CG: <MCID* Between Groups: †

Abbreviations: 4MWT, 4-meter walk test; 10-Meter Walk Test; CG, Comparison Group; IG, Intervention Group; IQR, interquartile range; NS, not Significant; RCT, randomized controlled trial; SS, statistically significant.

⁎ Statistically significant (p<0.05)

† Not statistically significant (p≥0.05)

#### Outcomes from variations of task-specific training

Outcomes from the studies that implemented variations of task-specific training are shown in table 7. Of the 5 studies, each study (20%) reported statistically significant improvements in one of the following measures: the 10MWT, 6MWT, and DGI. Additionally, 1 study (20%) reported improvement beyond the MCID on the 6MWT^51^ and beyond the MDC on the DGI.^53^

**Table 7:** Outcomes from variations of task-specific training.

Study	Design	Population	Treatment Protocol	Gait Assessment Methods	Outcomes
Hsieh et al, 2018^112^	Randomized crossover trial	IG (N=12): Age: 53.2±19.2 y Chonicity: Chonic (15.9±13.0 mo poststroke) CG (N=12): Age: 56.4±18.0 y Chonicity: Chonic (13.7±11.0 mo poststroke)	IG: 12 sessions of mirror therapy and functional task-specific training in the home over 4 wk, followed by a 4-wk washout period. Then, mirror therapy and functional task training were performed in a clinic setting for the same duration. CG: Same protocol as above in opposite order (clinical training, washout period, home training)	1) 10MWT	**10MWT:** IG: <MCID CG: <MCID Between Groups†
Jie et al, 2021^113^	RCT	IG (N=38) Age: 64.6±9.4 y Chonicity: Chonic (72.8±59.3 mo poststroke) CG (N=41) Age: 67.8±11.6 y Chonicity: Chonic (67.5±69.1 mo poststroke)	IG: Home gait training sessions using implicit learning methods; 9, 30-min sessions over 3 wk CG: Home gait training sessions using explicit learning methods; 9, 30-min sessions over 3 wk	1) 10MWT 2) DGI	**10MWT:** IG: <MCID CG: <MCID Between Groups † **DGI:** IG: >MDC CG: >MDC Between Groups: †
Malagoni et al, 2016^114^	RCT (pilot)	IG (N=6) Age: 62.5±13.8 y Chonicity: Chonic (6.2±3.5 y poststroke) CG (N=6) Age: 70.7±9.0 y Chonicity: Chonic (6.8±4.1 y poststroke)	IG: Personalized home walking program with metronome support; 6 d/wk for 10 wk CG: Clinic-based group rehabilitation, 3 times/wk for 10 wk	1) 6MWT	**6MWT:** IG: >MCID* CG: >MCID* Between Groups: †
Shaw et al, 2022^115^	RCT (pilot)	IG (N=30) Age: 71±10 y Chonicity: Chonic (312±159 d poststroke) CG (N=29) Age: 66±13 y Chonicity: Chonic (254±131 d poststroke)	IG: Gait and balance training using ARC; 3, 30-min sessions/wk for 6 wk CG: Gait and balance training without ARC; 3, 30-min sessions/wk for 6 wk	1) Gait speed (4MWT) 2) FAC	**Gait speed (4MWT):** IG: <MCID† CG: <MCID† **FAC:** IG: No change CG: 1 category improvement
Wright et al, 2017^116^	Single-group, within-subject crossover design (feasibility)	N=12 Age: 56±11 y Chonicity: Chonic (68±42 mo poststroke)	Two, 3-wk blocks of home-based stepping to music overlaid with metronome cues. Blocks consisted of standard and phase-shift perturbation tempos with a 3-wk washout. Training was self-administered 15 mins, 5 d/wk, with weekly researcher visits	1) 10MWT 2) DGI 3) Spatiotemporal asymmetry measures	**10MWT**: <MCID* DGI: <MDC* Spatiotemporal asymmetry measures: Step time asymmetry: <MDC† Swing time asymmetry: <MDC† (All measures after 2nd training block)

Abbreviations: 4MWT, 4-Meter Walk Test; 10MWT, 10-meter walk test; ARC, auditory rhythmic cueing; CG, Comparison Group; DGI, dynamic gait index; FAC, functional ambulation category; IG, Intervention Group; IQR, interquartile range; NS, Not Significant; RCT, randomized controlled trial; SS, Statistically Significant.

⁎ Statistically significant (p<0.05)

† Not statistically significant (p≥0.05)

#### Summary of outcomes

Although there was widespread variability in the approaches to treating gait dysfunction from home, a substantial proportion of studies reported statistically significant or clinically meaningful improvements in 1 or more gait-related outcomes. Specifically, such improvements were reported in 68% of studies using technology-integrated approaches, 75% of studies using home-use devices, 100% of studies using individualized home rehabilitation programs, 88% of studies incorporating self-management or coaching-based methods, 86% of studies involving trained nonclinicians to deliver home rehabilitation, and 60% of studies implementing task-specific training methodologies. These findings suggest that a range of home-based interventions may be effective in improving gait outcomes across different populations and delivery models.

## Discussion

This scoping review provides an exploratory overview of the peer-reviewed literature on home-based gait treatment for adults with stroke. The 62 studies included in this review demonstrate a variety of study designs, treatment methodologies, and assessments that explore the treatment of gait dysfunction, one of stroke’s most troubling consequences, from the home environment.

### Study characteristics

The included studies were conducted in 22 countries across the globe, highlighting broad interest in home-based rehabilitation for individuals with stroke. Notably, 60% of these studies were published in or after the year 2020, suggesting interest may have been accelerated by the global COVID-19 pandemic, which necessitated shifting clinical treatments to the home to ensure continuity of care.^15^ Despite this increase, each of the 6 gait treatment categories contains studies published both before and after the pandemic, indicating that the exploration of home-based gait rehabilitation was already underway before the pandemic’s onset.

Studies of all designs were eligible for inclusion in this review. However, although slightly over 50% of studies were randomized controlled trials, the substantial number of studies labeled as pilot, feasibility, preliminary, or proof-of-concept (39% of total studies) suggests that much of the research is in the exploratory stage. Although this scoping review did not evaluate the certainty or rigor of the evidence, the sizable number of preliminary investigations and single-group studies (40%) suggests the opportunity for more robust studies moving forward.

### Characteristics of the study population

Over 3000 adults with stroke participated in the studies included in this review. Both subacute and chronic stroke populations were represented, demonstrating the relevance of home-based gait treatment across varying stages of stroke recovery.

Notably, 73% of studies involved participants with a mean or median age less than 65, and none reported an average participant age over 75.^54^, ^55^, ^56^, ^57^, ^58^, ^59^, ^60^, ^61^, ^62^, ^63^, ^64^, ^65^, ^66^, ^67^, ^68^, ^69^, ^70^, ^71^, ^72^, ^73^, ^74^, ^75^, ^76^, ^77^, ^78^, ^79^, ^80^, ^81^, ^82^, ^83^, ^84^, ^85^, ^86^, ^87^, ^88^, ^89^, ^90^, ^91^, ^92^, ^93^, ^94^, ^95^, ^96^, ^97^, ^98^, ^99^, ^100^, ^101^, ^102^, ^103^, ^104^, ^105^, ^106^, ^107^, ^108^, ^109^, ^110^, ^111^, ^112^, ^113^, ^114^, ^115^, ^116^ Although the mean age of stroke onset has decreased in recent years,^117^ over 75% of strokes occur in individuals over 65, and 50% occur in those over 75 years of age.^118^ The relatively young participant population in this review may reflect several factors. Older stroke survivors are more likely to be institutionalized (thus less likely to be home-dwelling),^119^ and some older stroke survivors may have been excluded based on the cognitive requirements of some studies. Additionally, the emphasis on technology-integrated approaches may have been more suited to younger participants, a common finding among studies featuring technological approaches to rehabilitation care.^120^ Future studies should consider exploring feasibility, acceptability, and efficacy in older age groups more representative of the stroke population at large.

### Treatment approaches

An important finding of this review is the variety of treatment approaches used to address walking dysfunction in the home environment. Although some studies—particularly those categorized as “individualized home rehabilitation programs” and “variations of task-specific training”—used more traditional rehabilitation methods, such as strengthening, balance training, and gait-focused activities, over one-third of studies (21 studies, 34%) implemented technology-integrated approaches. These included mobile applications, gaming, VR-based programs, and digital platforms to boost enjoyment,^55^^,^^62^^,^^67^^,^^69^ monitor adherence,^57^^,^^63^^,^^72^^,^^73^ and/or improve access to rehabilitation.^65^^,^^67^^,^^71^ In addition to integrating these technological features, more than half of the studies (33 studies, 53%) used either partial or fully remote treatment delivery methods. The heavy presence of telehealth highlights a shift to virtual health care, including for gait rehabilitation, during and after the COVID-19 pandemic.

The second largest treatment category, home-use devices for gait treatment (12 studies, 19%), adapted clinic-based technologies for home-use. For example, Lopez-Rosado et al^82^ and Palmcrantz et al^84^ used electrical stimulation technologies embedded into wearable home-use garments; Prathum et al^86^ explored the use of transcranial direct current stimulation within the home setting, and Huizenga et al^81^ investigated the home application of a wearable device using split-belt treadmill principles. These approaches highlight a focus on innovation specifically designed for the home and underscore the need for future research that thoroughly investigates the therapeutic effect of these newer technologies.

The other treatment categories, “self-management or coaching-based programs” and “home rehabilitation delivered by trained nonclinicians,” focused on empowering patients or caregivers to administer rehabilitation without direct clinician involvement. For example, Chinchai and Khamwong^105^ trained village health workers to deliver home-based rehabilitation; Nordin et al^108^ explored the effectiveness of a home-based caregiver-assisted rehabilitation compared to hospital based, therapist-delivered treatment; and Preston et al^102^ implemented a personalized self-management program targeting physical activity and walking ability by teaching essential skills, such as goal setting, barrier identification, and self-monitoring. These studies illustrate strategies to extend clinical reach and promote independent mobility-supporting behaviors.

A comparison between the gait treatment approaches used in this review and those recommended in the literature reveals both alignment and notable differences. The Clinical Practice Guideline to Improve Locomotor Function Following Chronic Stroke, Incomplete Spinal Cord Injury, and Brain Injury^34^ recommends walking training at moderate to high intensities and virtual reality-based training to improve walking speed or distance based on strong, high-quality evidence. It also emphasizes that a large amount of task-specific training is critical for improvement in walking function, although only at higher cardiovascular intensities or with augmented feedback to increase patient engagement.^34^ Similarly, a 2022 narrative review on the current evidence for walking recovery after stroke emphasized the need for treatments that are tailored, task-oriented, and repetitive, with intensity as a key dosing parameter.^121^

Although various studies in this review incorporated task-specific practice, VR, and augmented feedback (see tables 2-7), few explicitly addressed treatment intensity. Although multiple studies (n=11) used accelerometers, pedometers, or other activity monitoring devices, only 2 studies^65^^,^^74^ measured heart rates during gait treatment. Given that recent research has demonstrated the feasibility of high-intensity home-based rehabilitation after stroke,^122^^,^^123^ measuring and targeting specific intensities during home-based gait treatment is worthy of future investigation.

### Assessments

The most frequently used assessments of gait function in this review were the 10MWT and the 6MWT. The frequent usage of these assessments is consistent with the gait rehabilitation literature, which recommends using these outcomes to assess changes in gait speed and walking distance.^46^ The Core Set of Outcome Measures for Adults With Neurologic Conditions clinical practice guideline also recommends using the FGA score; however, it was used in only three of the 62 studies.^46^

More than twice as many studies (34 studies compared 16 studies) measured gait treatment effects in clinical settings rather than the home. This was particularly evident in the 10 studies that measured spatiotemporal parameters of walking quality, such as step length or cadence. Despite recent studies that have indicated increased availability and accuracy of low-cost and portable sensor technologies,^39^ the usage of such equipment was low (3 studies).^59^^,^^79^^,^^116^ It has also been reported that the reliability of some outcomes may not translate to home settings. For example, Dunn et al^124^ found that variations of the standard 30-m walkway length for the 6MWT (typically required in homes because of spatial limitations) impacted the assessment’s accuracy. For patients seeking rehabilitation in their homes because of barriers or other factors limiting access to clinical environments, valid and reliable assessments of gait performance and capacity within the home are essential.

### Outcomes

Table 2, Table 3, Table 4, Table 5, Table 6, Table 7 summarize the outcomes reported within the 62 studies of this review. Because of the wide variety of treatments and large number of early-stage studies, this study does not critically appraise the individual studies or intend to identify the most effective methods for treating gait dysfunction. However, it was observed that more than 60% of individual studies within each gait treatment category reported statistically significant changes or clinically meaningful improvements on specific aspects of gait. Future reviews focused on particular study attributes, demographic characteristics, or desired outcomes could determine which treatments are most beneficial, under what circumstances, and in which contexts.

### The role of home-based care

This scoping review identified considerable variation in the implementation of home-based gait treatment across the studies. Interventions were delivered as standalone therapies (independent from other forms of therapy), as continuations of clinic-based rehabilitation (ie, after discharge from inpatient or outpatient care), or in combination with other treatment methodologies. These differences in timing and integration demonstrate the versatility of home-based approaches to meet rehabilitation needs at various stages of poststroke recovery. Future studies exploring the timing, duration, and long-term effects of home-based rehabilitation could help clarify how best to leverage the home setting to optimize gait outcomes after stroke.

Interestingly, despite the focus on home-based gait treatment, most studies still required participants to engage with clinical settings for introductory sessions, equipment training, or outcome assessments. The lack of fully home-based treatment paradigms may pose challenges for those with difficulty reaching clinical environments because of mobility impairments, geographic challenges, transportation difficulties, or other barriers. This finding underscores the need for further exploration into home-based approaches that are truly independent of clinical settings.

### Study limitations

This study aimed to thoroughly review the literature on home-based gait treatment for adults with stroke; however, several limitations were noted. First, the authors of the included studies were not contacted to provide additional study information, which may have clarified uncertain details, such as the location of gait assessments in 11 of the studies. Also, our literature search did not include hand-searching, which may have identified additional relevant articles. Lastly, given the exploratory nature of this scoping review, we did not critically appraise the individual sources of evidence. Therefore, outcome-related findings should be interpreted with appropriate caution.

## Conclusions

Given the recent increased interest in home-based rehabilitation, this scoping review sought to map the peer-reviewed literature regarding the methods and outcomes of home-based gait treatment for adults with stroke. Review of the literature revealed the global relevance of home treatment and a wide range of approaches to treating gait dysfunction within the home. A notable emphasis on technology integration and the innovative adaptation of clinical treatments to home-based methodologies was observed. This review also identified a substantial number of preliminary studies, indicating emerging interest in this field and highlighting the need for future rigorous investigations into treatment approaches, reliable home assessment methods, and therapeutic effectiveness. By synthesizing current practices and reported outcomes, this review underscores the need for future research that can help refine home-based interventions and ultimately enhance the gait outcomes for stroke survivors receiving treatment at home.

## Disclosure

The investigators have no financial or nonfinancial disclosures to make in relation to this project.

## Appendix 1 Search Strings

**Search Strings: September 12, 2024**

PubMed

("home*"[Title/Abstract] OR "home-based"[Title/Abstract] OR "house"[Title/Abstract] OR "residence"[Title/Abstract] OR "dwelling"[Title/Abstract] OR ("home care services, hospital based"[MeSH Terms] OR "Home Care Services"[MeSH Terms] OR "Hospital to Home Transition"[MeSH Terms])) AND ("Stroke"[MeSH Terms] OR "Brain Infarction"[MeSH Terms] OR "Stroke"[Title/Abstract] OR "ischemic attack, transient"[MeSH Terms] OR "cerebrovascular accident"[Title/Abstract] OR "CVA"[Title/Abstract] OR "brain infarct"[Title/Abstract] OR "transient ischemic attack"[Title/Abstract] OR "TIA"[Title/Abstract]) AND ("Gait"[MeSH Terms] OR "Walking"[MeSH Terms] OR "gait disorders, neurologic"[MeSH Terms] OR "Mobility Limitation"[MeSH Terms] OR "Gait"[Title/Abstract] OR "mobili*"[Title/Abstract] OR "ambulat*"[Title/Abstract] OR "locomot*"[Title/Abstract] OR "walk*"[Title/Abstract]) AND ("Therapeutics"[MeSH Terms] OR "treat*"[Title/Abstract] OR "therap*"[Title/Abstract] OR "intervention*"[Title/Abstract]) AND (y_10[Filter])

Cumulative Index to Nursing & Allied Health Literature (CINAHL)

((TI home* OR AB home*) OR (TI home-based OR AB home-based) OR (TI house OR AB house) OR (TI residence OR AB residence) OR (TI dwelling OR AB dwelling) OR ((MH "home care services, hospital based+") OR (MH "Home Care Services+") OR (MH "Hospital to Home Transition+"))) AND ((MH Stroke+) OR (MH "Brain Infarction+") OR (TI Stroke OR AB Stroke) OR (MH "ischemic attack, transient+") OR (TI "cerebrovascular accident" OR AB "cerebrovascular accident") OR (TI CVA OR AB CVA) OR (TI "brain infarct" OR AB "brain infarct") OR (TI "transient ischemic attack" OR AB "transient ischemic attack") OR (TI TIA OR AB TIA)) AND ((MH Gait+) OR (MH Walking+) OR (MH "gait disorders, neurologic+") OR (MH "Mobility Limitation+") OR (TI Gait OR AB Gait) OR (TI mobili* OR AB mobili*) OR (TI ambulat* OR AB ambulat*) OR (TI locomot* OR AB locomot*) OR (TI walk* OR AB walk*)) AND ((MH Therapeutics+) OR (TI treat* OR AB treat*) OR (TI therap* OR AB therap*) OR (TI intervention* OR AB intervention*))

• Added filter for publication range 2015 - present

Cochrane

(home*:ti,ab OR home-based:ti,ab OR house:ti,ab OR residence:ti,ab OR dwelling:ti,ab OR ([mh "home care services, hospital based"] OR [mh "Home Care Services"] OR [mh "Hospital to Home Transition"])) AND ([mh Stroke] OR [mh "Brain Infarction"] OR Stroke:ti,ab OR [mh "ischemic attack, transient"] OR "cerebrovascular accident":ti,ab OR CVA:ti,ab OR "brain infarct":ti,ab OR "transient ischemic attack":ti,ab OR TIA:ti,ab) AND ([mh Gait] OR [mh Walking] OR [mh "gait disorders, neurologic"] OR [mh "Mobility Limitation"] OR Gait:ti,ab OR mobili*:ti,ab OR ambulat*:ti,ab OR locomot*:ti,ab OR walk*:ti,ab) AND ([mh Therapeutics] OR treat*:ti,ab OR therap*:ti,ab OR intervention*:ti,ab)

• Applied limits for trials and 2015-present

Web of Science

(home* OR home-based OR house OR residence OR dwelling OR ("home care services, hospital based" OR "Home Care Services" OR "Hospital to Home Transition")) AND (Stroke OR "Brain Infarction" OR Stroke OR "ischemic attack, transient" OR "cerebrovascular accident" OR CVA OR "brain infarct" OR "transient ischemic attack" OR TIA) AND (Gait OR Walking OR "gait disorders, neurologic" OR "Mobility Limitation" OR Gait OR mobili* OR ambulat* OR locomot* OR walk*) AND (Therapeutics OR treat* OR therap* OR intervention*)

• Applied filter for publication years 2015-2024

Scopus

TITLE-ABS-KEY ((home* OR home-based OR house OR residence OR dwelling OR ("home care services, hospital based" OR "Home Care Services" OR "Hospital to Home Transition")) AND (stroke OR "Brain Infarction" OR stroke OR "ischemic attack, transient" OR "cerebrovascular accident" OR cva OR "brain infarct" OR "transient ischemic attack" OR tia) AND (gait OR walking OR "gait disorders, neurologic" OR "Mobility Limitation" OR gait OR mobili* OR ambulat* OR locomot* OR walk*) AND (therapeutics OR treat* OR therap* OR intervention*)) AND PUBYEAR > 2014 AND PUBYEAR < 2025

**Search Strings: January 10, 2025**

Pubmed

("home*"[Title/Abstract] OR "home-based"[Title/Abstract] OR "house"[Title/Abstract] OR "residence"[Title/Abstract] OR ("home care services, hospital based"[MeSH Terms] OR "Home Care Services"[MeSH Terms] OR "Hospital to Home Transition"[MeSH Terms])) AND ("Stroke"[MeSH Terms] OR "Brain Infarction"[MeSH Terms] OR "Stroke"[Title/Abstract] OR "ischemic attack, transient"[MeSH Terms] OR "cerebrovascular accident"[Title/Abstract] OR "CVA"[Title/Abstract] OR "brain infarct"[Title/Abstract] OR "transient ischemic attack"[Title/Abstract] OR "TIA"[Title/Abstract]) AND ("Gait"[MeSH Terms] OR "Walking"[MeSH Terms] OR "gait disorders, neurologic"[MeSH Terms] OR "Mobility Limitation"[MeSH Terms] OR "Gait"[Title/Abstract] OR "ambulat*"[Title/Abstract] OR "locomot*"[Title/Abstract] OR "walk*"[Title/Abstract]) AND ("Therapeutics"[MeSH Terms] OR "treat*"[Title/Abstract] OR "therap*"[Title/Abstract] OR "rehab*"[Title/Abstract] OR "train*"[Title/Abstract] OR "interven*"[Title/Abstract])

• Applied publication date filter 2024/09/13 to 2025/01/10

CINAHL

• Same as September 2024 search with custom range: 2024/09 to present

Cochrane

• Same as September 2024 search with custom range: 2024/09/13 to 2025/01/10

Web of Science

• Same as September 2024 search with publication filter for years 2024-2025

Scopus

• Same as September 2024 search with publication filter for years 2024-2025

## Appendix 2 Gait Assessments

**10-Meter Walk Test (10MWT)**: Assessment of walking speed^125^

**6-Minute Walk Test (6MWT)**: Measurement of walking endurance and aerobic capacity^126^

**Spatiotemporal Gait Parameters**: Measurements to assess spatial and temporal characteristics of walking. Spatial measurements include step length, stride length, and step width. Temporal measurements include step time, stride time, swing time, stance time, cadence, and double support time.^127^

**Functional Ambulation Categories (FAC)**: Broad characterization of walking ability that distinguishes six levels of walking based on the amount of physical support required.^128^

**Gait Subscale of the Tinetti Performance-Oriented Mobility Assessment (Tinetti Gait Assessment)**: Measurement of balance and walking ability. The gait subscale consists of 10 items assessing different aspects of walking.^129^

**Dynamic Gait Index (DGI)**: Evaluates functional stability during gait activities.^130^

**Functional Gait Assessment (FGA)**: Assessment of postural stability and ability to perform motor tasks during walking.^131^

**Kinematic and Kinetic Measurements**: Kinematic measurements describe body motions (such as position and joint angles) independently from the internal and external forces that cause them. Kinetic movements refer to the internal and external forces (such as ground reaction forces and joint torques) resulting in the observed movement patterns.^127^

**Gait Subscale of the Mini-BEST Test**: Assessment of the ability to maintain stability during different contexts of postural control. The gait subscale assesses stability during various walking tasks and the capacity to adjust gait in response to challenges.^132^

**Rivermead Visual Gait Assessment**: Visual assessment of gait quality.^133^

**Gait Subscale of the Brunel Balance Assessment**: The Brunel Balance Assessment is a hierarchical measure of balance disability poststroke. Section 3, which can be scored separately, focuses on stepping-based tasks.^134^

**Four Square Step Test**: Clinical test of standing balance and gait which involves four-directional stepping over low objects.^135^

**Figure 8 Walking**: Walking assessment which combines curved-path walking and navigation to assess the abilities necessary for independence in daily walking activities.^136^

**Modified Emory Functional Ambulation Profile**: Gait assessment tool which measures time to ambulate through five common environmental terrains.^137^

**GaitRite Functional Ambulation Profile**: Measurement of spatial and temporal variables of gait obtained using a pressure sensor system walkway.^138^
